# Growing up with a single mother and life satisfaction in adulthood: A test of mediating and moderating factors

**DOI:** 10.1371/journal.pone.0179639

**Published:** 2017-06-15

**Authors:** David Richter, Sakari Lemola

**Affiliations:** 1 German Institute for Economic Research, Berlin, Germany; 2 Department of Psychology, University of Warwick, Coventry, United Kingdom; TNO, NETHERLANDS

## Abstract

Single parenthood is increasingly common in Western societies but only little is known about its long-term effects. We therefore studied life satisfaction among 641 individuals (ages 18–66 years) who spent their entire childhood with a single mother, 1539 individuals who spent part of their childhood with both parents but then experienced parental separation, and 21,943 individuals who grew up with both parents. Individuals who grew up with a single mother for their entire childhood and to a lesser degree also individuals who experienced parental separation showed a small but persistent decrease in life satisfaction into old age controlling childhood socio-economic status. This decrease was partly mediated by worse adulthood living conditions related to socio-economic and educational success, physical health, social integration, and romantic relationship outcomes. No moderation by age, gender, and societal system where the childhood was spent (i.e. western oriented FRG or socialist GDR) was found.

## Introduction

Single parenthood is increasingly common in Western societies, with 27.5% of children in the US currently being raised in single-parent households—more than 80% of them in households headed by single mothers [1]. Although the importance of studying the long-term consequences of single parenthood on children is clear, there is still a dearth of knowledge on the relative strength of long-term effects of single parenthood on children’s well-being at different stages of the adult life-span as well as on the involved mechanisms. Therefore, we study differences in life-satisfaction across adulthood related to differences in childhood family structure in a large representative German panel study. We focus on life-satisfaction in adulthood as a highly desirable characteristic which is assumed to play a crucial role for the populations' health, longevity, and citizenship [2, 3].

There are three main pathways by which being raised by a single mother may produce a long-lasting impact on well-being in adulthood. First, children in single-mother households are more likely to suffer from less effective guardianship and a higher likelihood of family distress and conflicts (e.g., [4]). It is well established that two-parent families generally provide more emotional resources to children than single-parent families (e.g., [5, 6]). In a related vein, children, whose parents divorce, exhibit slightly lower psychological well-being and social adjustment than children from stable two-parent families (e.g., [5, 7, 8–10]). The experience of parental divorce may cause further emotional distress to the child [5, 11] and may eventually lead to an insecure attachment representation [5, 12]. Prolonged family distress and insecure attachment representation may in turn complicate the development of social skills and make it more difficult to engage in satisfying intimate relationships which may eventually also hamper life-satisfaction during adulthood [12].

A second pathway of impact is related to the generally lower socio-economic status and increased risk of economic deprivation among children in single-mother households (e.g., [4]). Economic deprivation affects children's adjustment and well-being in multiple ways. Children from poor households are at increased risk to live in a low quality home environment and poor neighborhood conditions. They are more often exposed to harsh parental rearing practices and poor parental mental health, and they more often receive suboptimal nutrition and suffer from poor physical health [13]. Finally, economic deprivation also increases the likelihood of these children to enter careers with poor socio-economic prospects and to show poor social integration when they reach early adulthood [5].

A third pathway can be summarized as the ‘missing-father hypothesis.’ In popular science, it has been discussed that children need both a mother and a father, presuming that fathering involves distinct and necessary qualities which are particularly important for gender identity formation in boys (e.g., [14, 15]). There is also evidence that the absence of a father is associated with an increase in antisocial behaviors in boys, including violence, criminality, and substance abuse [16] and a decrease in social adjustment in general [5].

### The present study

In the present study, we examine whether general life satisfaction is lower among adults raised by a single mother than for adults raised in two-parent families. To do so, we compare the general life satisfaction of adults reared by their single mothers with respondents who grew up with both parents. As single parenthood and parental divorce are associated with parental socio-economic background and education, we statistically control for parents’ education and occupational prestige along with the respondents’ age and sex.

We expect to find a dose-response relationship, that is, that adults who spent at least part of their childhood in a two-parent family are affected less—despite the significant stresses associated with the experience of parental separation [5]. We expect a smaller decrease in general life satisfaction in this group, as the parent who left the family may still provide resources to support children when they enter adulthood—which is less likely when the parent has never lived together with the child.

Second, we test mediation models namely whether the association between childhood family structure and general adulthood life satisfaction is mediated by life outcomes that may be summarized as adulthood life success, including educational attainment, employment status, occupational prestige, net income, physical health, integration into social networks, and success in romantic relationships as there is evidence that these life-circumstances are affected in a negative way by growing up in a single parent household and/or by having experienced parental divorce [5]. We hypothesize that differences in these life circumstances during adulthood partly explain the difference in general adulthood life satisfaction between individuals who have been raised by single mothers and their counterparts who grew up with both parents.

Third, we test moderation of the effects by three possible moderating variables, age, gender, and societal system where the children grew up. Regarding age differences one might assume that the effects of single parenthood wane across the adult life-span following the general psychological principle that the longer ago a negative experience the smaller the imposed impact (e.g., [17]). Regarding gender differences we test the idea frequently echoed in popular science, namely that men who were raised by single mothers are more disadvantaged in adulthood than their female counterparts. Finally, regarding the question if different societal systems differentially affect the role of childhood family settings for adulthood life satisfaction we compare individuals who grew up in the Federal Republic of Germany and in the German Democratic Republic. The western oriented Federal Republic of Germany (FRG) and the socialist German Democratic Republic (GDR), which existed between 1949 and 1990, differed sharply in terms of several variables that may possibly be relevant for single parent families namely divorce rate, female participation in the labor market, and child day-care infrastructure. The divorce rate in the socialist GDR was nearly twice as high as in the FRG and female participation in the labor market was at 89% compared to 55% in the FRG in 1990 [18]. Even more drastic difference existed with regard to the child day-care infrastructure; more than half of the children who grew up in the socialist GDR were in regular day-care, which was free of charge, while less than 2% were in day-care in the FRG at the end of the 1980s [19]. Due to these differences we expect that children who grew up with single mothers in the socialist GDR were less disadvantaged compared to their counterparts who grew up with both parents than children who grew up with single mothers in the FRG; we expect this, as the higher divorce-rate may have reduced the stigma associated with single parenthood in the GDR, moreover, single motherhood was possibly related with relatively less economic burden in the GDR compared to the FRG.

## Methods

### Sample

The data are from the SOEP (Version 30), which is an ongoing, nationally representative longitudinal study of private households in Germany running since 1984. Comprehensive information about the data collection, design, respondents, variables, and assessment procedures is reported in Wagner, Frick, and Schupp [20].

The sample comprised of 26,936 adults born after 1946, of whom 24,123 adults between the ages of 17 and 66 years (*M* = 37.86 years, *SD* = 13.50 years; 52.1% female) were analyzed in the present paper. Given the present study’s focus on the effect of single parenthood vs. growing up with both parents, we categorized the participants into three subgroups: individuals who lived with both parents up to the age of 15 (*n* = 21,943), those whose parents separated and who lived with their mothers for between one and fourteen years (*n* = 1539), and those who lived with a single mother up to the age of 15 (*n* = 641). Data from 2813 respondents were excluded who had spent part of their childhood in different family settings (e.g., raised by the mother and a new partner, by a single father with or without a new partner, or by other relatives; among the excluded respondents there were 207 individuals who grew up with a single father for 1–14 years and 21 individuals who grew up with a single father for 15 years, respectively).

Regarding the societal system where the children grew up, in the FRG, 18,186 respondents grew up with both parents up to the age of fifteen, 1234 lived with their mothers for between one and fourteen years, and 483 lived with a single mother up to the age of fifteen. In the former GDR, 3757 respondents grew up with both parents up to the age of fifteen, 305 lived with their mothers for between one and fourteen years, and 158 lived with a single mother up to the age of fifteen.

### Materials

Although life satisfaction has been measured since the very beginning of the SOEP study in 1984, the information on where respondents had spent the first fifteen years of their lives was only available for respondents who entered the panel after the year 2000. During the fourteen years of data collection, respondents reported their general life satisfaction (‘All things considered, how satisfied are you with your life in general?’) at the end of each yearly interview using an 11-point scale ranging from 0 (*completely dissatisfied*) to 10 (*completely satisfied*), a measure with high reported reliability and validity [21]. To minimize error variance and to get a global indicator of adult well-being, general life satisfaction was estimated by aggregating all data available to build a mean-score (*M* = 7.33, *SD* = 1.49). On average, respondents provided 4.71 (*SD* = 4.29; *range* = 1–14) data points of general life satisfaction.

When entering the panel study, respondents reported where they had grown up in the first fifteen years of their life (“How many years of your childhood (up until age fifteen) did you live with the following persons? Please round off to the nearest year”). For our analyses, we used data from the response options “with both your father and mother (biological or adoptive)” and “with your mother without a new husband or partner”.

The participants also reported their socio-economic status (SES) in childhood (i.e., their parents’ education and occupational prestige), their own SES in adulthood (i.e., employment status, occupational prestige, education, and net income), their physical health status during adulthood (the number of visits to the doctor, reverse-coded), their social integration in adulthood (number of friends, number of visits to/from friends, and number of visits to/from family members), and success in romantic relationships (their relationship status and if they were divorced). Descriptive statistics of the study variables for the three subgroups are presented in Table 1.

**Table 1:** Descriptive statistics of study variables by childhood family settings.

	Both Parents 15y	Single Mother 1-14y	Single Mother 15y	Linear trend
*N*	21,943	1539	641	
Age ^a^	38.19 (13.44) ^c^	33.80 (13.27) ^d^	36.53 (14.38) ^e^	*p* < .01
Sex (*% female*)	51.88	54.78	53.35	
Life satisfaction ^a^	7.35 (1.48) ^c^	7.21 (1.55) ^d^	7.07 (1.66) ^d^	*p* < .001
Father’s education ^b^				
• Unknown	29.39	30.28	56.79	
• Low	42.13	38.60	22.62	
• High	28.48	31.12	20.59	
Father’s occupational prestige ^a^	34.38 (17.12) ^c^	27.91 (17.59) ^d^	22.55 (15.02) ^e^	*p* < .001
Mother’s education ^b^				
• Unknown	29.68	29.50	35.26	
• Low	41.95	38.01	41.97	
• High	28.37	32.49	22.78	
Mother’s occupational prestige ^a^	26.31 (16.44) ^c^	27.86 (16.87) ^d^	24.31 (14.86) ^e^	*p* < .01
Education ^b^				
• Low	20.10	29.30	33.70	
• Medium	52.82	49.38	51.17	
• High	27.07	21.31	15.13	
Occupational prestige ^a^	36.36 (16.84) ^c^	33.75 (16.52) ^d^	31.15 (15.66) ^e^	*p* < .001
Employment status ^a^	0.55 (0.40) ^c^	0.48 (0.40) ^d^	0.45 (0.41) ^d^	*p* < .001
Monthly net income € ^a^	1166.74(1280.40) ^c^	942.21 (1066.64) ^d^	806.33 (895.67) ^d^	*p* < .001
Doctor visits ^a^	8.10 (9.86) ^c^	8.60 (11.03) ^c^	8.34 (10.55) ^c^	*p* = .605
Number of friends ^a^	4.62 (3.67) ^c^	4.32 (3.05) ^d^	4.08 (3.15) ^d^	*p* < .01
Visits to/from friends ^a^	3.36 (0.83) ^c^	3.45 (0.88) ^d^	3.41 (0.92) ^d^	*p* = .242
Visits to/from family ^a^	3.27 (0.87) ^c^	3.25 (0.90) ^c^	3.20 (1.02) ^c^	*p* = .130
Partnership status ^a^	0.64 (0.46) ^c^	0.53 (0.48) ^d^	0.55 (0.47) ^d^	*p* < .001
Divorced ^a^	0.02 (0.11) ^c^	0.02 (0.11) ^c^	0.03 (0.12) ^c^	*p* = .376

^a^ *M*, *SD* in brackets.

^b^ in percent. Values with different superscripts

^(c, d, e)^ vary significantly (*p* < 0.05; Bonferroni-corrected).

Life satisfaction: Scale range 0 (completely dissatisfied) to 10 (completely satisfied);

Occupational prestige: Standard International Occupation Prestige Score index (SIOPS), Scale range 13–78; Employment status: 1 = full-time, 0.5 = regular part-time/vocational training, 0.25 = irregular part-time, 0 = not employed; Doctor visits per year; Visits to/from friends and Visits to/from family: Scale range 1 (daily) to 5 (never), reversed; Partnership status: 1 = in a partner relationship, 0 = not in a partner relationship; Divorced: 1 = divorced, 0 = not divorced.

Occupational prestige was scored from 13 to 78 using the Standard International Occupation Prestige Score index (SIOPS; [22]). Occupational prestige was not available for 5377 (22.3%) of the respondents and for 12,331 (51.1%) mothers and 7097 (29.4%) fathers of respondents. In most cases these individuals had no occupational prestige due to being homemakers or being unemployed. In rare cases, however, participants also did not know their parents’ occupation. Missing occupational prestige was scored with the lowest value possible following the rationale that being unemployed or homemaker is regarded as lower in prestige than all other paid work. Respondents’ general occupational prestige was estimated by calculating the mean of all yearly data available.

Education of parents measured when respondents entered the panel and scored from 1 to 3 (no education [1]: no school attendance, no degree obtained, other degree obtained, or respondent did not know; low education [2]: lower-track secondary school; and high education [3]: intermediate-track or upper-track secondary school). Education of respondents was scored using the International Standard Classification of Education (ISCED-1997; [23]. Prior to the analyses respondents’ ISCED-Scores were collapsed into three categories (low education [1]: ISCED-Scores 0, 1, and 2; medium education [2]: ISCED-Scores 3 and 4; and high education [3]: ISCED-Scores 5 and 6). Missing information on education (*n* = 138, 0.6%) was scored as the lowest category.

Yearly data on the employment status of respondents were coded to generate a continuous index (full-time employment was coded 1.0, regular part-time employment or vocational training were coded 0.5, marginal, irregular part-time employment was coded 0.25, and not employed was coded 0.0) and collapsed into a mean score to represent the general employments status of respondents across the years they reported their life satisfaction.

The number of doctor visits as well as their generalized monthly net income in EUR were estimated by calculating the mean of all yearly data available.

Social network status was measured in the years 2003, 2008, and 2013. Respondents reported how often they “visited or were visited by neighbors, friends, or acquaintances” and how often they “visited or were visited by family members or relatives” on a 1 (*daily*) to 5 (*never*) scale. In the analysis, the scales of these variables were reversed. In addition, respondents answered the question “how many close friends would you say that you have?”. Respondents’ general social network status was estimated by calculating the mean of all data available.

Respondents’ partnership status was coded (with partner was coded 1.0, no partner was coded 0.0) and collapsed into a mean score to represent the respondent’s general relationship status across the years they reported their life satisfaction. Similarly, we coded whether respondents’ marital status was “divorced” (divorced was coded 1.0, all other marital statuses were coded 0.0) for the years they reported their life satisfaction and collapsed the data into a mean score.

Intercorrelations of all study variables are depicted in S1 Table.

### Analyses

In a first step, respondents’ z-standardized general life satisfaction served as the dependent variable in hierarchical multiple regression analyses. In this analysis, dummy-coded variables were used to represent the childhood family settings of the subgroups. These analyses controlled respondents’ age, age^2^, age^3^, and sex as well as parents’ education (dummy coded) and parents’ occupational prestige (standardized). Age was centered before age^2^ and age^3^ were calculated.

In a second step, analyses of variance were conducted to test whether indicators of adulthood life outcomes including adulthood SES, physical health, social integration, and success in romantic relationships varied significantly in the three aforementioned subgroups. Again, respondents’ age, age^2^, age^3^, and sex as well as parents’ education (dummy coded) and occupational prestige (standardized) were entered into the equations to control for these background variables.

In a third step, mediation analyses were conducted to test whether differences in adulthood life satisfaction related to childhood family structure were mediated by indicators of adulthood life outcomes including adulthood SES, physical health, social integration, and success in romantic relationships in adulthood. These possible mediators of the effect of childhood family settings on general life satisfaction were entered in three blocks. In model 1 (baseline model), parents’ education (dummy coded) and occupational prestige (standardized) were included into the equation to control for childhood SES. In model 2, respondents’ own education (dummy coded), occupational prestige (standardized), employment status (centered), and net income (standardized) were entered as one block representing adulthood SES. In model 3, respondents’ adulthood physical health (number of doctor visits, reverse coded, and centered) was entered to the equation. Finally, in model 4 respondents’ number of friends (centered), visits to/from friends (centered), visits to/from family members (centered), partnership status (centered), and having been divorced (centered) were entered as one block representing adulthood social integration and success in romantic relationships.

First, we compared the variance explained by childhood family settings (only controlling age, age^2^, age^3^, and sex) with the variance that childhood family settings explained after the control variables of model 1 (childhood SES) had been entered to the regression model. Second, we compared the variance explained by childhood family settings in model 1 (only controlling childhood SES) with the variance that childhood family settings explained after the mediators of model 2 (adulthood SES) had been entered to the regression model. Third, we compared the variance explained by the childhood family settings in model 2 with the variance that childhood family settings explained after the mediators of model 3 (model 2 mediators plus physical health) had been entered to the regression model. Finally, we compared the variance explained by the childhood family settings in model 3 with the variance that childhood family settings explained after the mediators of model 4 (model 3 mediators plus adulthood social integration and success in romantic relationships) had been entered to the regression model.

Additionally, we also evaluated indirect paths of childhood family settings on adulthood general life satisfaction via these mediators employing the Structural Equation Modeling module of stata 13. Here, all possible indirect paths were tested in individual models controlling age, age^2^, age^3^, sex, and childhood SES.

In a fourth step, we included interaction terms into the regression analyses to analyze if the effects of the childhood family structure on adulthood life satisfaction varied depending on respondents’ sex and age when completing the questionnaire following the procedure proposed by Aiken and West [24]. In addition, we tested whether associations of the different childhood family settings with general life satisfaction in adulthood differed for individuals who grew up in the FRG or the GDR.

The analyses were conducted with SPSS 20 and stata 13.

## Results

### Childhood family settings and adulthood life satisfaction

**Fig 1:**
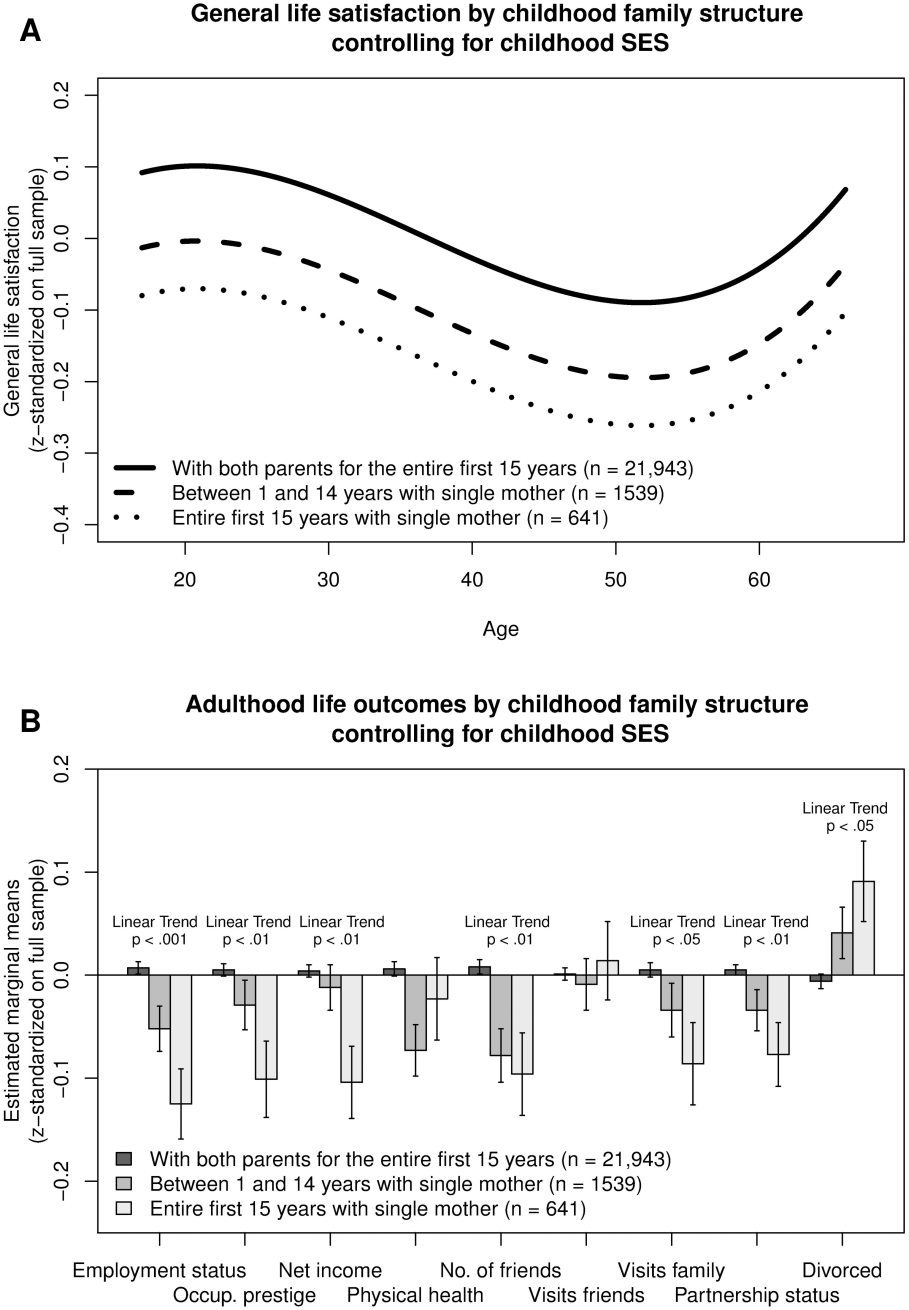
A. Association of general life satisfaction with childhood family settings across the adult life-span controlling for respondents’ sex and childhood SES. 1B. Association of adulthood life outcomes (adulthood SES, physical health, social integration, and romantic relationship success) with childhood family settings controlling for respondents’ sex, age, and childhood SES.

The main analyses showed a significant association of the different childhood family settings with general life satisfaction. Compared to people raised by both parents, respondents reared by a single mother for between 1 and 14 years or for the entire first 15 years of their lives reported significantly lower general life satisfaction than the group reared by both parents. The effect sizes for the difference in life satisfaction between the two groups reared by a single mother and the group reared by both parents were in the small range (1–14 years: *d* = 0.10 *p* < .001, entire first 15 years: *d* = 0.19, *p* < .001). Fig 1A depicts the association between childhood family settings and adulthood life satisfaction across the adult life-span controlling for childhood SES. The values underlying Fig 1A are reported in Table 2, Model 1. The association between childhood family settings and adulthood life satisfaction was not moderated by respondents’ age or respondents’ sex (for further details see below).

**Table 2:** Unstandardized regression coefficients (B) and intercept for variables as predictor of general life satisfaction.

Dependent Variable:	Zero-Order Effects	Model			
General Life Satisfaction		1	2	3	4
Intercept	-	-.009	-.054***	-.038*	-.103***
*Years reared by a single mother*					
1–14 years	-.095***	-.105***	-.096***	-.086***	-.069**
15 years	-.186***	-.172***	-.136***	-.135***	-.108**
Age	-.093***	-.092***	-.145***	-.131***	-.126***
Age^2^	.006	.006	.059***	.056***	.095***
Age^3^	.012***	.013***	.010**	.011***	-.005
Sex (0 = *female*, 1 = *male*)	-.045***	-.048***	-.183***	-.202***	-.194***
*Father’s education (ref*.: *low)*					
Unknown	.019	-.016	.002	.001	.004
High	.098***	.009	-.023	-.024	-.034
Father’s occupational prestige	.039***	.038***	.017*	.018*	.021*
*Mother’s education (ref*.: *low)*					
Unknown	.046**	.055*	.090***	.084***	.077***
High	.121***	.048*	.025	.021	.017
Mother’s occupational prestige	.023***	-.014	-.010	-.012	-.008
*Education (ref*.: *medium)*					
Low	-.026		-.057**	-.050**	-.051**
High	.185***		.099***	.095***	.090***
Occupational prestige	.127***		.037***	.039***	.039***
Employment status	.245***		.186***	.151***	.167***
Net income	.127***		.125***	.123***	.108***
Physical health	.018***			.016***	.016***
Number of Friends	.035***				.025***
Visits to/from friends	.166***				.115***
Visits to/from family	.085***				.043***
Partnership status	.169***				.379***
Divorced	-.884***				-.402***
Observations		24,123	24,123	24,123	24,123
Adjusted *R*^2^		.009***	.048***	.068***	.105***

We report unstandardized coefficients. The dependent variable was standardized before analysis. Values for age are given in 10-year units. Age was centered before higher order terms were calculated. Education of respondents and respondent’s parents were dummy coded. Occupational prestige and net income were standardized; employment status, physical health (number of doctor visits, reverse coded), number of friends, visits to/from friends and family, partnership status, and having been divorced were centered.

* *p* < .05.

** *p* < .01.

*** *p* < .001.

### Childhood family settings and adulthood life circumstances

Fig 1B depicts the various domains of adult life outcomes including adulthood SES, physical health, adulthood social integration, and romantic relationship success separately for individuals who grew up with both parents, who lived with a single mother for between one and 14 years (i.e., individuals whose parents separated at some point in childhood), or who spent their first 15 years living with a single mother, controlling for childhood SES. Growing up with a single mother was associated with lower SES in childhood including lower parental education and occupational prestige (mother’s education *p* < .01, all other *p*s < .001). Growing up with a single mother was further related to the participants’ own SES in adulthood including employment status, occupational prestige, and net income. This association exhibited evidence of a dose-response relationship: individuals who spent their first 15 years living with a single mother reported lower SES in adulthood than individuals who spent between 1 and 14 years living with a single mother, who again were lower than their counterparts who lived with both parents throughout childhood, controlling for their childhood SES (all linear trends *p* < 0.05).

Participants who spent their first 15 years with a single mother further showed a lower degree of social integration during adulthood, including a smaller number of friends and fewer visits to/from family as well as less success in romantic relationships, including a lower probability of living with a partner and a higher probability of having been divorced, controlling for childhood SES (linear trends *p* < 0.05). Again the effect was somewhat stronger for participants who lived with a single mother for their first 15 years compared to their counterparts whose parents separated at some point during childhood. Generally, the effect sizes were in the modest range, and no significant association between childhood family settings and physical health (number of doctor visits, reverse-coded) and number of visits to/from friends was revealed after controlling childhood SES (see also S2 Table).

### Mediation of the effect on life satisfaction by adulthood life circumstances

Mediation analyses revealed that a large part of the variance in life satisfaction between different childhood family settings was explained by childhood SES, including differences in the education and occupational prestige of the respondents’ parents (i.e., 29% of the variance; see Table 2, Model 1). Inclusion of respondents’ own education, occupational prestige, employment status, and net income during adulthood into the model attenuated the association of the different childhood family settings with general life satisfaction by a further 20% (Model 2). Inclusion of physical health (Model 3) attenuated the association of the different childhood family settings with life satisfaction by a further 6%. Finally, inclusion of respondents’ social integration and success in romantic relationships attenuated the association of the different childhood family settings with life satisfaction by a further 16% (Model 4). However, the differences in general life satisfaction between respondents who lived with both parents for their first 15 years of life and either group of respondents reared by a single mother remained significant in all models, even when all adulthood life circumstances were controlled for.

**Fig 2:**
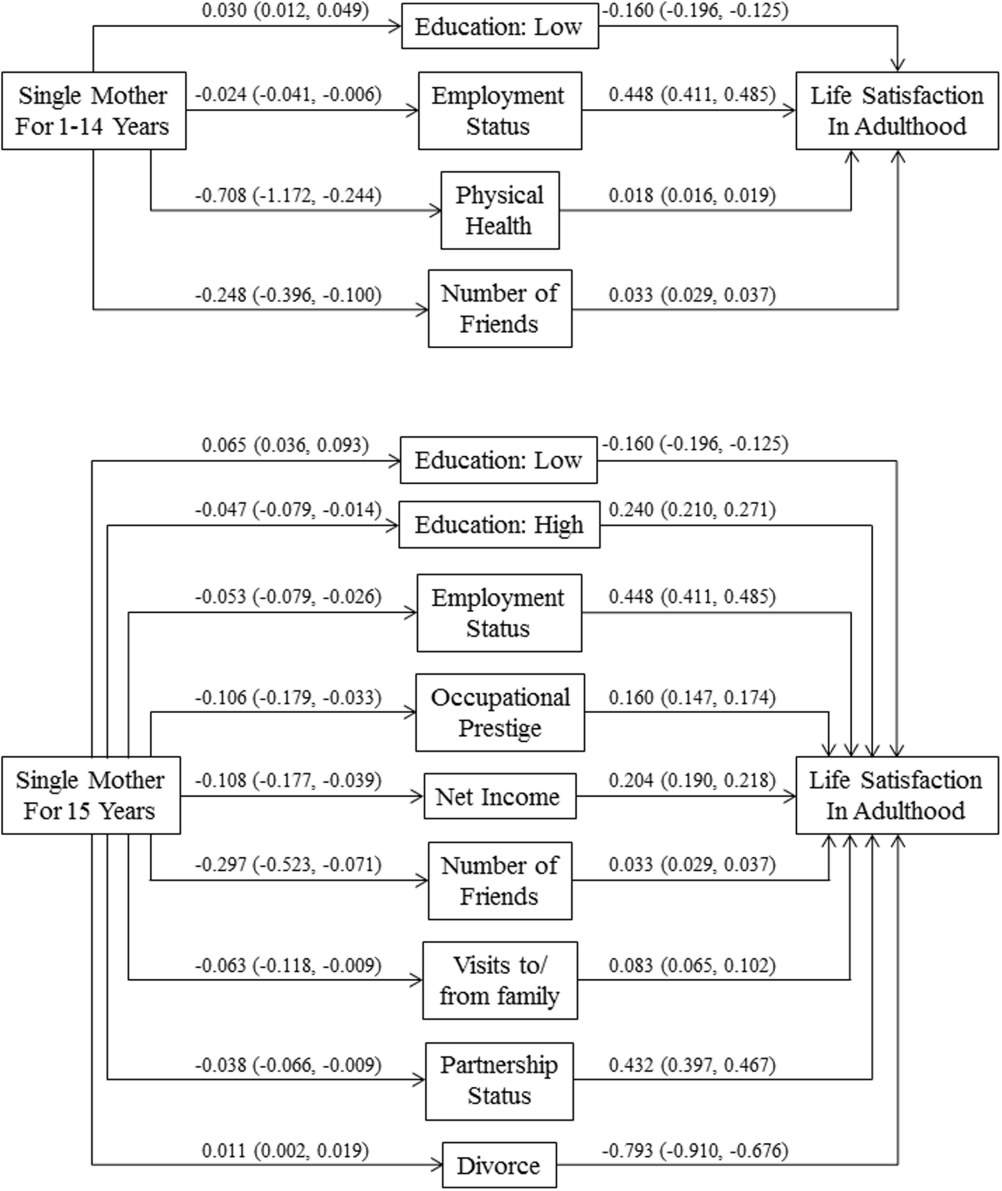
Results of path model estimating indirect effects from childhood family settings to adult life-satisfaction via adulthood life circumstances. Indirect paths were estimated separately in individual models but illustrated here together in one model for presentational parsimony. All models controlled age, age2, age3, sex, and childhood SES. Values are unstandardized path coefficients with 95% confidence limits. Life satisfaction, occupational prestige and net income were standardized; employment status, physical health (number of doctor visits, reverse coded), number of friends, visits to/from family, partnership status, and having been divorced were centered.

Evaluation of the indirect paths between ‘growing up with a single mother for 1–14 years vs. with both parents’ and general life satisfaction revealed that paths mediated by respondents’ education, employment status, physical health, and number of friends were significant (p < 0.05, see Fig 2). Regarding indirect paths between ‘growing up with a single mother for the entire childhood vs. with both parents’ and general life satisfaction, paths mediated by respondents’ education, employment status, occupational prestige, net income, number of friends, visits to/from family, partnership status, and experience of divorce in adulthood were significant (p < 0.05, see Fig 2).

### Moderation of the effect of life circumstances on life satisfaction by sex

Testing sex differences regarding the role of these adulthood life circumstances for life satisfaction revealed that physical health (i.e., the reverse-coded number of doctor visits; men: *β* = .09, *t* = 2.46, *p* > .05, women: *β* = .20, *t* = 5.80, *p* < .001, sex × physical health interaction: *t* = 2.66, *p* < .01) and number of friends (men: *β* = .05, *t* = 1.17, *p* = .241, women: *β* = .16, *t* = 4.61, *p* < .001, sex × number of friends interaction: *t* = 2.54, *p* < .01) were more strongly associated with life satisfaction among women who spent between 1 and 14 years of their childhood living with a single mother when compared to their male counterparts. No respective interactions with sex were found for those who spent 15 years living with a single mother.

### Moderation of the effect of childhood family settings on life satisfaction by age, sex, and societal system (FGR vs. GDR)

Moderation effects of the association between childhood family settings and adulthood life satisfaction by respondents’ age and respondents’ sex were non-significant when controlling for respondents’ childhood SES (age: *F*(6, 24104) = 0.807, *p* = .564, all age × years with single mother interactions: *t* < 0.45, *p* > .656; sex: *F*(2, 24108) = 2.554, *p* = .078, sex × 1–14 years with single mother interaction: *t* = 1.74, *p* = .081, sex × 15 years with single mother interaction: *t* = 1.51, *p* = .131), indicating that the effect does not change with age and does not differ between men and women. In addition, the association between childhood family settings and adulthood life satisfaction did not differ significantly between individuals who grew up in the FGR or the GDR (*F*(2, 24107) = 0.734, *p* = .480, Societal System × 1–14 years with single mother interaction: *t* = 1.14, *p* = .253, Societal System × 15 years with single mother interaction: *t* = 0.34, *p* = .731). This effect remained non-significant (*F*(2, 13687) = 0.834, *p* = .453) when the sample was restricted to individuals born between 1946 and 1974 who lived for their whole childhood until the age of fifteen in the FRG or GDR, respectively.

## Discussion

This is the first study to show that growing up with a single mother is related to a stable although modest reduction in general life satisfaction across the adult life-span until old age when adjusting for poor childhood SES. Individuals who spent their entire first 15 years of life living with a single mother showed on average approximately twice the reduction in life satisfaction compared to individuals who spent only part of their first 15 years with a single mother, which is consistent with a dose-response relationship. This suggests that growing up with a single mother throughout all of childhood and early adolescence and the related lack of resources from the father more than outweighs the well-described negative effects related to parental separation [5, 7–9].

The reduction in adulthood life satisfaction was partially mediated by the individuals’ living conditions, including their lower socio-economic status and educational level, lower physical health status, and poor social integration and romantic success in adulthood. This finding is consistent with studies on adult well-being after parental divorce [5, 25]. The decrease in adulthood life satisfaction was not moderated by age, thus we could not find waning of the effect of single parenthood with increasing distance to childhood. This is in contrast to evidence on negative life events during adulthood including divorce, bereavement, and unemployment for which the general principle of adaptation holds positing that the impact of an negative event decreases with increasing time since the event has happened (e.g., [17, 26]). However, and in contrast to studies on effects of negative life events during adulthood we here studied long-term effects of enduring childhood family settings which are possibly more likely to lead to long-term changes to the set-point of general life-satisfaction during adulthood. Moreover, we could not find evidence supporting the widely held notion from popular science that boys are more affected than girls by the absence of their fathers. However, we did find that in females who experienced parental separation during childhood, the effect was more strongly mediated by poor physical health and a smaller number of friends than in their male counterparts.

Finally, we did not find evidence for differential associations between growing up with a single mother in the western oriented FRG compared to the socialist GDR––this although one might expect that the higher divorce rate in the GDR could have reduced the stigma associated with single parenthood in the GDR. Moreover, one might expect that the higher rate of female participation in the work force as well as the higher number of children in day-care in the socialist GDR might have mitigated inequalities between children raised in single parent households compared to children from two-parent households in the GDR.

However, our finding of a non-significant difference between the FRG and the GDR is consistent with comparisons between children raised by single parents in states with well-established welfare systems such as Norway as compared to children from single parents from states with less well-established welfare systems such as the US who neither found any differences [27]. One explanation for the lack of differences in such comparisons can be summarized by a relative deprivation perspective which holds that existing small economic differences may still matter a lot in societies with a more even distribution of goods and which is in contrast to an absolute economic deprivation perspective [26]. A second explanation for finding no differences between the FRG and the GDR is that our respondents who grew up in the GDR responded to the study many years after the breakdown of the socialist state of the GDR in 1990. The breakdown of the socialist system has lead to many changes and new economic hardships to a part of the population [28]. It remains possible that such economic hardships might have stroke adults who grew up with a single mother more strongly than their counterparts who grew up in two-parent families as they possibly also received less support from their father while they were already adults. A third explanation for finding no differences between the FRG and the GDR is that the socio-emotional resources provided by the father were also lacking in single-parent households in the GDR. The deprivation from the father's socio-emotional resources may have outbalanced the effects of some possibly more favorable societal circumstances for single-parents in the GDR.

As a limitation of the study, it remains impossible to derive causality as growing up in a single-mother household and adulthood life satisfaction might both be influenced by a third variable such as genetic factors. In this respect, there is evidence that the risk of divorce is up to 30–40% hereditary which is mediated by personality traits such as negative affectivity [29]. In a similar vein, it is possible that the direction of the causal influence between the factors that we tested as mediators and life satisfaction are different than we have specified them. For instance it is possible that the relationship between physical health and life satisfaction is reverse involving an impact of life satisfaction on physical health.

A further limitation lies in the measurement of the childhood family settings which were reported retrospectively during adulthood. While it may be assumed that adults are able to reliably report whether they spent the entire childhood vs. only a part of their childhood with a single mother, this variable may still be subject to memory distortions. Furthermore, regarding the possible mediating factors of the effect of childhood family settings on adulthood life satisfaction, physical health could have been measured in a more sophisticated fashion. In the present study it was assessed by the number of visits to the doctor, while more objective measures of physical health such as a doctor’s examinations or physical fitness tests might have revealed different findings.

In conclusion, the present study shows that growing up with a single mother—in particular if the father is absent for the entire childhood—predicts a small but stable decrease in life satisfaction across adulthood that is partly explained by lower socio-economic status and educational achievement, inferior physical health, poor social integration, and lower likelihood of romantic relationship success in adulthood. Contrary to expectations this effect was not moderated by sex, age, or the societal system in which the childhood was spent. Thus, the differences in life satisfaction were similar for younger and older, male and female, as well as participants who spent their childhood in the western oriented FRG or in the socialistic GDR.

Future cross-cultural research comparing effects of family settings on adulthood life-outcomes in several studies from different cultures may identify macro-level protective factors that could be targeted to improve the prospects of single parents and their children.

## Supporting information

S1 Table Intercorrelations of study variables. * p < .05. ** p < .01. *** p < .001.
(DOCX)

S2 Table Estimated marginal means of adulthood life circumstances by childhood family settings controlling participants' sex, age, and childhood SES *(z-standardized on full sample; M*, *SE in brackets)*. Values with different superscripts vary significantly (*p* < 0.05; Bonferroni-corrected).
(DOCX)

S1 File SPSS-Syntax of the main analyses. Stata-syntax of the mediation analyses. Those not using SPSS or stata may check the included output-file.
(ZIP)
