# Supplementary material for: Growing up with a single mother and life satisfaction in adulthood: A test of mediating and moderating factors
Source: PLoS One. 2017 Jun 15;12(6):e0179639. doi: 10.1371/journal.pone.0179639 (PMC5472317; doi:10.1371/journal.pone.0179639)
Supplement: S1 Table — * p < .05. ** p < .01. *** p < .001. (DOCX) [file pone.0179639.s001.docx]

|  |  | 1 | 2 | 3 | 4 | 5 | 6 | 7 | 8 |
| --- | --- | --- | --- | --- | --- | --- | --- | --- | --- |
| (1) | 1-14 years with single mother | - |  |  |  |  |  |  |  |
| (2) | 15 years with single mother | -.043*** | - |  |  |  |  |  |  |
| (3) | Life satisfaction | -.022*** | -.029*** | - |  |  |  |  |  |
| (4) | Age | -.079*** | -.016* | -.068*** | - |  |  |  |  |
| (5) | Sex (0 = *female,* 1 = *male*) | -.014* | -.004 | -.022*** | -.002 | - |  |  |  |
|  | Father’s education |  |  |  |  |  |  |  |  |
| (6) | Unknown | .001 | .096*** | -.010 | -.067*** | -.012 | - |  |  |
| (7) | Low | -.015* | -.063*** | -.028*** | .270*** | .002 | -.552*** | - |  |
| (8) | High | .016* | -.029*** | .041*** | -.227*** | .010 | -.414*** | -.530*** | - |
| (9) | Father’s occupational prestige | -.087*** | -.106*** | .039*** | -.021** | .012 | -.519*** | .057*** | .466*** |
|  | Mother’s education |  |  |  |  |  |  |  |  |
| (10) | Unknown | -.002 | .020* | -.001 | -.054*** | -.003 | .789*** | -.428*** | -.336*** |
| (11) | Low | -.020* | .001 | -.041*** | .330*** | -.006 | -.440*** | .640*** | -.252*** |
| (12) | High | .023*** | -.021** | .046*** | -.305*** | .009 | -.320*** | -.266*** | .616*** |
| (13) | Mother’s occupational prestige | .024*** | -.021** | .023*** | -.218*** | -.002 | -.310*** | -.036*** | .355*** |
|  | Education |  |  |  |  |  |  |  |  |
| (14) | Low | .053*** | .051*** | -.036*** | -.277*** | -.017 | .177*** | -.110*** | -.060*** |
| (15) | Medium | -.017** | -.005 | -.046*** | .057*** | -.037*** | -.046*** | .131*** | -.097*** |
| (16) | High | -.030*** | -.042*** | .085*** | .192*** | .057*** | -.112*** | -.047*** | .165*** |
| (17) | Occupational prestige | -.036*** | -.048*** | .127*** | .123*** | .098*** | -.201*** | .061*** | .138*** |
| (18) | Employment status | -.045*** | -.040*** | .098*** | .228*** | .303*** | -.063*** | .098*** | -.043*** |
| (19) | Net income | -.042*** | -.044*** | .127*** | .273*** | .301*** | -.133*** | .080*** | .049*** |
| (20) | Physical health | -.011 | -.003 | .161*** | -.122*** | .099*** | .009 | -.042*** | .037*** |
| (21) | Number of Friends | -.015* | -.017** | .101*** | -.068*** | .036*** | -.023*** | -.045*** | .072*** |
| (22) | Visits to/from friends | .021** | .006 | .109*** | -.289*** | -.016* | .000 | -.107*** | .117*** |
| (23) | Visits to/from family | -.005 | -.009 | .059*** | -.019** | -.061*** | .013* | .042*** | -.059*** |
| (24) | Partnership status | -.059*** | -.028*** | .078*** | .517*** | -.019** | .013* | .124*** | -.148*** |
| (25) | Divorced | -.004 | .006 | -.096*** | .155*** | -.028*** | -.054*** | .077*** | -.030*** |

|  |  | 9 | 10 | 11 | 12 | 13 | 14 | 15 | 16 |
| --- | --- | --- | --- | --- | --- | --- | --- | --- | --- |
| (1) | 1-14 years with single mother |  |  |  |  |  |  |  |  |
| (2) | 15 years with single mother |  |  |  |  |  |  |  |  |
| (3) | Life satisfaction |  |  |  |  |  |  |  |  |
| (4) | Age |  |  |  |  |  |  |  |  |
| (5) | Sex (0 = *female,* 1 = *male*) |  |  |  |  |  |  |  |  |
|  | Father’s education |  |  |  |  |  |  |  |  |
| (6) | Unknown |  |  |  |  |  |  |  |  |
| (7) | Low |  |  |  |  |  |  |  |  |
| (8) | High |  |  |  |  |  |  |  |  |
| (9) | Father’s occupational prestige | - |  |  |  |  |  |  |  |
|  | Mother’s education |  |  |  |  |  |  |  |  |
| (10) | Unknown | -.478*** | - |  |  |  |  |  |  |
| (11) | Low | .101*** | -.551*** | - |  |  |  |  |  |
| (12) | High | .374*** | -.411*** | -.534*** | - |  |  |  |  |
| (13) | Mother’s occupational prestige | .457*** | -.343*** | -.063*** | .417*** | - |  |  |  |
|  | Education |  |  |  |  |  |  |  |  |
| (14) | Low | -.138*** | .177*** | -.133*** | -.034*** | -.039*** | - |  |  |
| (15) | Medium | -.053*** | -.048*** | .124*** | -.087*** | -.020** | -.544*** | - |  |
| (16) | High | .187*** | -.110*** | -.018** | .131*** | .059*** | -.309*** | -.630*** | - |
| (17) | Occupational prestige | .210*** | -.204*** | .086*** | .113*** | .081*** | -.326*** | -.129*** | .448*** |
| (18) | Employment status | .016* | -.061*** | .124*** | -.073*** | -.059*** | -.290*** | .032*** | .233*** |
| (19) | Net income | .120*** | -.124*** | .110*** | .005 | -.024*** | -.268*** | -.129*** | .393*** |
| (20) | Physical health | .008 | .009 | -.054*** | .049*** | .037*** | .001 | -.009 | .009 |
| (21) | Number of Friends | .043*** | -.015* | -.052*** | .072*** | .053*** | .000 | -.025*** | .028*** |
| (22) | Visits to/from friends | .038*** | .001 | -.130*** | .141*** | .083*** | .079*** | -.032*** | -.037*** |
| (23) | Visits to/from family | -.041*** | .005 | .045*** | -.054*** | -.028*** | .004 | .047*** | -.058*** |
| (24) | Partnership status | -.062*** | .022*** | .165*** | -.202*** | -.178*** | -.250*** | .058*** | .166*** |
| (25) | Divorced | .037*** | -.054*** | .092*** | -.046*** | -.001 | -.048*** | .029*** | .011 |
|  |  | 17 | 18 | 19 | 20 | 21 | 22 | 23 | 24 |
| (1) | 1-14 years with single mother |  |  |  |  |  |  |  |  |
| (2) | 15 years with single mother |  |  |  |  |  |  |  |  |
| (3) | Life satisfaction |  |  |  |  |  |  |  |  |
| (4) | Age |  |  |  |  |  |  |  |  |
| (5) | Sex (0 = *female,* 1 = *male*) |  |  |  |  |  |  |  |  |
|  | Father’s education |  |  |  |  |  |  |  |  |
| (6) | Unknown |  |  |  |  |  |  |  |  |
| (7) | Low |  |  |  |  |  |  |  |  |
| (8) | High |  |  |  |  |  |  |  |  |
| (9) | Father’s occupational prestige |  |  |  |  |  |  |  |  |
|  | Mother’s education |  |  |  |  |  |  |  |  |
| (10) | Unknown |  |  |  |  |  |  |  |  |
| (11) | Low |  |  |  |  |  |  |  |  |
| (12) | High |  |  |  |  |  |  |  |  |
| (13) | Mother’s occupational prestige |  |  |  |  |  |  |  |  |
|  | Education |  |  |  |  |  |  |  |  |
| (14) | Low |  |  |  |  |  |  |  |  |
| (15) | Medium |  |  |  |  |  |  |  |  |
| (16) | High |  |  |  |  |  |  |  |  |
| (17) | Occupational prestige | - |  |  |  |  |  |  |  |
| (18) | Employment status | .609*** | - |  |  |  |  |  |  |
| (19) | Net income | .597*** | .664*** | - |  |  |  |  |  |
| (20) | Physical health | .041*** | .078*** | .048*** | - |  |  |  |  |
| (21) | Number of Friends | .025*** | -.020** | -.001 | -.021** | - |  |  |  |
| (22) | Visits to/from friends | -.030*** | -.120*** | -.094*** | -.036*** | .223*** | - |  |  |
| (23) | Visits to/from family | -.030*** | -.004 | -.040*** | .010 | .048*** | .244*** | - |  |
| (24) | Partnership status | .150*** | .261*** | .258*** | .044*** | -.071*** | -.215*** | .048*** | - |
| (25) | Divorced | .042*** | .061*** | .052*** | .034*** | -.054*** | -.075*** | -.027*** | -.104*** |
