# Supplementary material for: Growing up with a single mother and life satisfaction in adulthood: A test of mediating and moderating factors
Source: PLoS One. 2017 Jun 15;12(6):e0179639. doi: 10.1371/journal.pone.0179639 (PMC5472317; doi:10.1371/journal.pone.0179639)
Supplement: S2 Table — Values with different superscripts vary significantly (p < 0.05; Bonferroni-corrected). (DOCX) [file pone.0179639.s002.docx]

|  | Both Parents 15y | Single Mother  1-14y | Single Mother 15y | Linear trend |
| --- | --- | --- | --- | --- |
| Employment status | 0.007 (0.006)^a^ | -0.052 (0.022)^b^ | -0.125 (0.034)^b^ | *p* < .001 |
| Occupational prestige | 0.005 (0.006)^a^ | -0.029 (0.024)^a,b^ | -0.101 (0.037)^b^ | *p* < .01 |
| Net income | 0.004 (0.006)^a^ | -0.012 (0.022)^a,b^ | -0.104 (0.035)^b^ | *p* < .01 |
| Physical health | 0.006 (0.007)^a^ | -0.073 (0.025)^b^ | -0.023 (0.040)^a,b^ | *p* = .475 |
| No. of friends | 0.008 (0.007)^a^ | -0.078 (0.026)^b^ | -0.096 (0.040)^b^ | *p* < .01 |
| Visits to/from friends | 0.001 (0.006)^a^ | -0.009 (0.025)^a^ | 0.014 (0.038)^a^ | *p* = .714 |
| Visits to/from family | 0.005 (0.007)^a^ | -0.034 (0.026)^a^ | -0.086 (0.040)^a^ | *p* < .05 |
| Partnership status | 0.005 (0.005)^a^ | -0.034 (0.020)^a,b^ | -0.077 (0.031)^b^ | *p* < .01 |
| Divorced | -0.006 (0.007)^a^ | 0.041 (0.025)^a,b^ | 0.091 (0. 039)^b^ | *p* < .05 |
| *N* | 21,943 | 1539 | 641 |  |
